# Fragility Fractures in Orthopaedics: An Update

**DOI:** 10.1155/2017/1924069

**Published:** 2017-01-19

**Authors:** Zhiyong Hou, Cyril Mauffrey, Wade R. Smith, Marius M. Scarlat

**Affiliations:** ^1^Department of Orthopaedic Surgery, Third Hospital of Hebei Medical University, Shijiazhuang, Hebei 050051, China; ^2^Department of Orthopaedics, Denver Health Medical Center, University of Colorado School of Medicine, Denver, CO 80204, USA; ^3^Mountain Orthopaedic Trauma Surgeons at Swedish, 701 East Hampden Avenue, Suite 515, Englewood, CO 80113, USA; ^4^Chirurgie Orthopédique et Traumatologique, Clinique Chirurgicale St Michel, Toulon, France

Osteoporosis is the most common metabolic bone disorder, and its incidence increases with age. It is predicted that in 2050 there will be a large explosion in the elderly population in China, with up to 400 million aged 65+ (26.9% of the total population) and 150 million aged 80+ [1]. Due to the osteoporotic change of bone, a significant number of osteoporotic patients experience fragility fractures. There were reports that fragility fractures account for close to 2 million fractures annually in United States and could increase to more than 3 million by 2025 [2]. Owing to the high morbidity and mortality, fragile fractures significantly decrease the survival quality of the elderly [3]. Additionally, the fragile bone tissue increases the difficulty of operative fixation. Thus, fragility fractures present challenges for orthopaedic surgeons in the modern era. Up till now, there has been a continuous effort in improving the prevention and treatment for fragile fractures. Therefore, we create this special issue to provide better understanding of fragility fractures.

This special issue covers a variety of articles focused on the appealing topics of fragility fractures. These original researches from different countries include risk factor analysis, epidemiologic research, imaging diagnosis, and clinical study.

Fragility fractures may occur with relatively small energy of trauma, and fall related fractures are very important health issue in this more aging society. In one research article, H.-Y. Pi et al. tried to identify the risk factors of those fractures and effective preventive measures and found that dementia that emerged is the most important risk factor for the in-hospital complications of fall-related fractures, while most of the preventive measurements could not reduce the incidence of the in-hospital complications. Fragility fractures associated with osteoporosis may also be caused by thyroid disease. In this issue, G. Maccagnano et al. defined the prevalence of fragility fractures in relation to thyroid disease in a region of southern Italy and confirmed the role of thyroid disease as a risk factor in the onset of fragility fractures. Since patients receiving glucocorticoid are at a higher risk of developing secondary osteoporosis, assessment of bone microarchitecture may be used to evaluate risk of fragility fractures of osteoporosis. Within this issue, a pre-post study of M.-H. Chuang et al. declared that trabecular bone score (TBS) and fracture risk assessment tool (FRAX) adjusted with TBS (T-FRAX) showed an amplified predictive ability in the evaluation of risk of fragility fractures in patients receiving glucocorticoid therapy.

As the ageing trend is predicted to continue [4], new treatment strategies have been popularised for managing fractures associated with osteoporosis, including the use of new devices and surgical skills. A. Ortega-Briones et al. reported that column fixation and simultaneous total hip arthroplasty is a viable option for complex geriatric acetabular fractures and as well showed excellent mid-term results. Vertebral compression fractures are one of the most common fragility fractures in osteoporotic patients [5]. B.-S. Chang et al. suggested that anterior spinal column reconstruction using femoral shaft allografts improved kyphosis and resulted in minimal subsidence and therefore recommended it as an effective treatment option for dealing with osteoporotic vertebral collapse and kyphotic deformity. Surgical treatment of proximal humeral fractures in osteoporotic bone of elderly patients is challenging. Through the analysis of the clinical and radiological outcome, R. Bogner et al. recommended the use of Humerusblock device for the treatment of two- and three-part proximal humeral fractures. Locking plate has made a great progress in treating displaced proximal humeral fractures, which makes the joint-preserving surgery method more successful in elderly osteoporotic patients [6]. Given the characteristics of osteoporosis, L. Jin et al. introduced a probing method with depth gauge to determine the proximal screw length, which can make the screw-tip adjoin the subchondral bone and keep the articular surface of humeral head intact and at the same time effectively avoid frequent X-ray fluoroscopy and adjusting the screws.

In summary, fragile fractures are always challenging the orthopaedic surgeons. We have selected some valuable articles in this special issue. In the future, continuous efforts will be made to seek optimal prevention and treatment strategies in this field.
